# The effect of lifestyle interventions on maternal body composition during pregnancy in developing countries: a systematic review

**DOI:** 10.5830/CVJA-2017-003

**Published:** 2017

**Authors:** D Watson Estelle, J-L Gradidge Philippe, D Watson Estelle, Macaulay Shelley, Macaulay Shelley, Lamont Kim, J Crowther Nigel, Libhaber Elena

**Affiliations:** Centre for Exercise Science and Sports Medicine, School of Therapeutic Sciences, Faculty of Health Sciences, University of the Witwatersrand, Johannesburg, South Africa; Centre for Exercise Science and Sports Medicine, School of Therapeutic Sciences, Faculty of Health Sciences, University of the Witwatersrand, Johannesburg, South Africa; MRC/Wits Developmental Pathways of Health Research Unit, Department of Paediatrics, School of Clinical Medicine, Faculty of Health Sciences, University of the Witwatersrand, Johannesburg, South Africa; MRC/Wits Developmental Pathways of Health Research Unit, Department of Paediatrics, School of Clinical Medicine, Faculty of Health Sciences, University of the Witwatersrand, Johannesburg, South Africa; Division of Human Genetics, School of Pathology, Facultyof Health Sciences, University of the Witwatersrand andthe National Health Laboratory Service, Johannesburg,South Africa; Soweto Cardiovascular Research Unit, University of the Witwatersrand, Johannesburg, South Africa; Department of Chemical Pathology, National Heath Laboratory Service, Faculty of Health Sciences, University of the Witwatersrand, Johannesburg, South Africa; School of Clinical Medicine, Faculty of Health Sciences, University of the Witwatersrand, Johannesburg, South Africa

**Keywords:** diet, physical activity, pregnancy, obesity, gestational weight gain

## Abstract

Optimal maternal body composition during pregnancy is a public health priority due to its implications on maternal health and infant development. We therefore aimed to conduct a systematic review of randomised, controlled trials, and case–control and cohort studies using lifestyle interventions to improve body composition in developing countries. Of the 1 708 articles that were searched, seven studies, representing three countries (Brazil, Iran and Argentina), were included in the review. Two articles suggested that intervention with physical activity during pregnancy may significantly reduce maternal weight gain, and five studies were scored as being of poor quality. This systematic review highlights the lack of research within developing countries on lifestyle interventions for the management of excessive weight gain during pregnancy. Similar reviews from developed countries demonstrate the efficacy of such interventions, which should be confirmed using well-designed studies with appropriate intervention methods in resource-limited environments.

## Abstract

Both developed and developing countries are experiencing a rapid increase in the prevalence of obesity, which places affected individuals at an increased risk for a number of different diseases, including hypertension, diabetes, heart disease, asthma and cancer.^1^,^2^ The World Health Organisation (WHO) estimated that in 2005 there were approximately 1.6 billion adults (aged 15 years and over) globally who were overweight and at least 400 million adults who were obese.^3^

Especially alarming is the high prevalence of overweight and obesity among women of childbearing age in both developed and developing countries. Around 12 to 38% of pregnant women in developed countries,^5^ and 8 to 26% of pregnant women in developing countries4,6 are reported to be overweight or obese.

Obesity during pregnancy is associated with an increased risk for maternal and neonatal complications. The associated adverse maternal effects of obesity during pregnancy include miscarriage, pre-eclampsia, gestational diabetes mellitus, infection, venous thromboembolism and haemorrhage.^7^ The foetal risks associated with maternal obesity include stillbirths and neonatal deaths, preterm births, congenital abnormalities and macrosomia.^8^ Long-term effects of maternal obesity on the offspring have also been observed and include increased risks of childhood and adolescent obesity, and diabetes and cardiovascular disease in adult life.^9^

Both under- and overweight pose a risk to the mother and child during and after birth.^10^ It is therefore important to carefully manage weight gain during pregnancy with dietary intake of a sufficient level to ensure proper foetal nutrition,^11^ but avoiding excessive maternal weight gain. The use of lifestyle interventions to attenuate such weight gain during pregnancy has been the focus of many studies in the developed world, with a recent systematic review of 88 studies, involving 182 139 women, showing that maternal weight control during pregnancy via diet, exercise or a mix of these methods is safe and improves both maternal and foetal outcomes.^12^ However, no similar analysis of such data from the developing world is currently available.

The health risks of maternal obesity and excessive gestational weight gain to the mother and baby pose significant demands on the healthcare system, with an increased need for additional resources in both primary and secondary care settings.^11^,^13^ This is particularly true in developing countries where insufficient resources exist to meet these extra demands on the public health system, and where obesity is already prevalent. It is therefore important to develop cost-effective interventions to reduce maternal obesity in such environments. In an attempt to determine the effectiveness of maternal lifestyle interventions in resource-limited environments, we conducted a systematic review of the literature on weight-management protocols for pregnant females, undertaken in developing countries.

## Methods

Five electronic databases were searched; these included Public/ Publisher MEDLINE (PubMed), SCOPUS, a bibliographic database containing abstracts and citations for academic journal articles, Biomed Central, the Cochrane Library and the Cumulative Index to Nursing and Allied Health (CINHAL). Twenty-four search terms, with varying combinations, encompassing pregnancy, obesity/overweight, diet/nutrition, physical activity and developing countries (Table 1), were used. The search included all articles published up to 4 December 2013. No filters were set, in order to obtain articles in all languages and all types of documents.

**Table 1:** Search terms

Search 1	Pregnancy AND obesity AND diet AND developing countries
Search 2	Pregnancy AND obesity AND nutrition AND developing countries
Search 3	Pregnancy AND obesity AND physical activity AND developing Countries
Search 4	Pregnancy AND obesity AND exercise AND developing countries
Search 5	Pregnancy AND overweight AND diet AND developing countries
Search 6	Pregnancy AND overweight AND nutrition AND developing Countries
Search 7	Pregnancy AND overweight AND physical activity AND developing Countries
Search 8	Pregnancy AND overweight AND exercise AND developing countries
Search 9	Pregnancy AND obesity AND diet AND middle-income countries
Search 10	Pregnancy AND obesity AND nutrition AND middle-income countries
Search 11	Pregnancy AND obesity AND physical activity AND middle-income Countries
Search 12	Pregnancy AND obesity AND exercise AND middle-income Countries
Search 13	Pregnancy AND overweight AND diet AND middle-income Countries
Search 14	Pregnancy AND overweight AND nutrition AND middle-income Countries
Search 15	Pregnancy AND overweight AND physical activity AND middle income Countries
Search 16	Pregnancy AND overweight AND exercise AND middle-income Countries
Search 17	Pregnancy AND obesity AND diet AND low-income countries
Search 18	Pregnancy AND obesity AND nutrition AND low-income countries
Search 19	Pregnancy AND obesity AND physical activity AND low-income countries
Search 20	Pregnancy AND obesity AND exercise AND low-income countries
Search 21	Pregnancy AND overweight AND diet AND low-income countries
Search 22	Pregnancy AND overweight AND nutrition AND low-income countries
Search 23	Pregnancy AND overweight AND physical activity AND low-income countries
Search 24	Pregnancy AND overweight AND exercise AND low-income countries

Randomised, controlled trials (RCTs), case–control studies and cohort studies that were performed in developing countries and which investigated overweight/obesity in pregnant women and/or lifestyle interventions during pregnancy were considered eligible. Investigations performed in developing countries and the following types of studies were not eligible for inclusion: reviews, position statements/guidelines, reports, epidemiological studies, observational studies and prevalence studies.

The literature search was performed independently by two authors, Shelley Macaulay (SM) and Estelle Watson (EW). The search results obtained from each of the five electronic databases were pooled and duplicates were removed. At the first step, titles were screened for eligibility. Following this, the abstracts of those that were considered eligible were then obtained and read. Fulltext articles of the abstracts that fulfilled the inclusion criteria were then obtained and read. In addition, the reference lists of appropriate full-text articles were hand-searched for further relevant articles.

Data were extracted from the full-text articles by four of the reviewers: Phillipe Gradidge (PG), Elena Libhaber (EL), EW and SM. For each included article, data were extracted for country, region, sample size, gestational age, BMI, intervention details and outcomes.

The quality of each included article was assessed by three authors (EW, PG and EL) using the Cochrane Risk of Bias Tool (Cochrane, 2011). In accordance with the risk-assessment checklist, each study was assessed on: sequence generation, allocation concealment, blinding, incomplete outcome data, selective outcome reporting and other threats to validity.

The studies were classified as being good, average or poor quality based on how many of the above criteria were met. Good-quality articles met five or more of the above criteria, average-quality articles met three to four of the above criteria, and poor-quality ones met less than three of the above criteria.

## Results

**Fig. 1:**
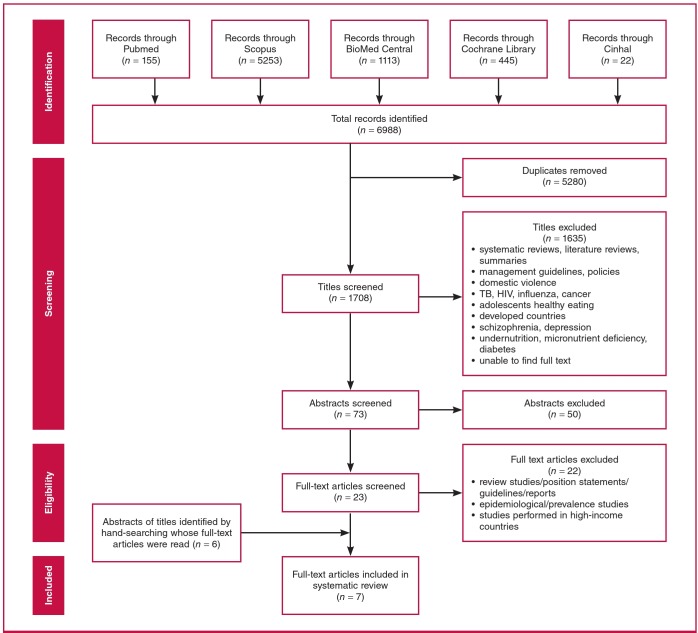
Flow diagram illustrating the number of included and excluded studies in the systematic review on lifestyle interventions for obesity/overweight during pregnancy in developing countries

A total of 6 988 records were identified from the five databases, after which 5 280 duplicates were removed. The title screen therefore involved 1 708 articles, of which 73 were considered appropriate, and their abstracts were obtained and reviewed. After reviewing the abstracts, 23 full-text articles were obtained and read. In addition, the bibliography of the full-text articles were hand-searched for further appropriate articles. Six additional articles were obtained through hand-searching. Together with the hand-searched articles, a final total of seven articles were considered eligible for this systematic review. Articles that were excluded at this stage were those conducted in high-income countries and articles involving women post delivery of their babies (Fig. 1).

The results of the Cochrane Risk of Bias Tool are displayed in Table 2, highlighting the criteria for assessing quality and risk of bias. Two of the included six articles displayed adequate sequence generation, allocation concealment, and addressed incomplete outcome data.^14^,^15^ However, only one of these reported a low risk of bias for ‘blinding’ of participants.^14^ The final outcome of the quality assessment showed that five out of the seven articles (71%) were of poor quality.

**Table 2:** Reporting quality and risk-of-bias assessment

	Cochrane tool for assessing bias						
*Author*	*Sequence Generation*	*Allocation Concealment*	*Blinding*	*Incomplete*	*Selective outcome Reporting*	*Other threats to Validity*	*Final outcome*
Santos et al., 2005^14^	Yes	Yes	Yes	Yes	Unclear	Yes	Good
Sedaghati et al., 2007^16^	No	No	No	Yes	Unclear	No	Poor
Garshasbi et al., 2005^19^	Unclear	Unclear	No	Yes	Unclear	Yes	Poor
Prevedel et al., 2003^17^	Unclear	Unclear	No	No	Yes	Yes	Poor
Cavalcante et al., 2009^18^	Yes	Yes	No	Yes	Yes	Yes	Good
Malpeli et al., 2013^11,^	No	No	No	Yes	Yes	No	Poor
Ghodsi & Asltoghiri, 2012^15^	No	No	No	Yes	Unclear	Yes	Poor

The articles comprised five RCTs from Brazil and Iran and two non-RCTs from Argentina and Iran, as shown in Table 3.^11^,^14^-^19^ Characteristics of the interventions across the seven studies are detailed in Table 4. Six of the trials studied the impact of exercise alone on maternal and birth outcomes, and one study investigated using fortified food to enhance micronutrient nutritional status.

A study by Prevedel et al.^17^ was one of two that used aquatic physical activity as an intervention. The relative body fat percentage of the experimental group remained at 29%, however, the control group increased by 1.9%.

**Table 3:** Details and characteristics of the final studies included in the systematic review

*Author*	*Type of Study*	*Country*	*Region*	*Study setting (urban/rural)*	*Inclusion criteria*	*Sample size*	*Age (years)*	*Gestational age (weeks)*
Santos et al., 2005^14^	RCT	Brazil	Porto Alegre	Public health clinic (not specified)	Healthy, non-smoking, ≥ 20 years, gestational age < 20 weeks; BMI 26–31 kg/m^2^	Control 35 Exercise 37	Control 28.6 ± 5.9 Exercise 26.0 ± 3.4	Control 18.4 ± 3.9 Exercise 17.5 ± 3.3
Garshasbi et al., 2005^19^	RCT	Iran	Tehran	Hospital	Primi-gravid, 20–28 years old, 17–22 weeks’ gestation, housewives, high-school graduated	Control 105 Exercise 107	Control 26.48 ± 4.43 Exercise 26.27 ± 4.87	Not specified
Malpeli et al., 2013^11^	Nonrandomised	Argentina	Buenos Aires	Urban	Sample from lowincome families, pregnant women, without chronic or infectious diseases	Control 164 Experimental 108	Control 25.8 ± 6.4 Intervention 26.3 ± 7.1	Control 23.6 ± 9.3 Intervention 24.3 ± 8.00
Sedaghati et al., 2007^16^	Nonrandomised	Iran	Qom Province	Pre-natal clinics	Exclusion: history of orthopaedic diseases or surgery, history of exercise before pregnancy	Control 50 Experimental 40	Control 23.36 ± 4.237 Exercise 23.28 ± 2.522	Control 38.884 ± 1.232 Exercise 39.195 ± 0.921
Prevedel et al., 2003^17^	RCT	Brazil	Sao Paulo	Pre-natal clinic of the Faculty of Medicine de Botucatu (urban)	Primi-gravid or adolescents, singleton pregnancies, no co-morbidities	Intervention 22 Control 19	Mean: 20 years	16–20
Cavalcante et al., 2009^18^	RCT	Brazil	Sao Paulo	Pre-natal outpatient clinic of the University of Campinas and the neighbouring basic healthcare centre	Low-risk, sedentary pregnant women who had not had more than 1 C-section and were able to participate in physical exercise	Intervention 34 Control 34	Not specified	16–20
Ghodsi & Asltoghiri, 2012^15^	RCT	Iran	Unspecified	Pre-natal clinics and hospitals	BMI 19.8–26 kg/m2; lack of specific disease, willingness to participate, correct address for follow up; ability to read and write; nulliand primi-gravid	Total sample 250; unclear on specific numbers in each group	Control 25.86 ± 4.90 Training 25.43 ± 4.52	20–26

RCT = randomised, control trial; BMI = body mass index.

**Table 4:** Details and characteristics of the interventions in the studies

*Author*	*Type of study*	*Intervention(type)*	*Intervention (details)*	*Intervention (duration)*	*Outcome measure*	*Pre-pregnancy BMI/weight*	*Weight/BMI*	*Conclusion*
Santos et al., 2005^14^	RCT	Supervised PA	60 min, 3 days/week, 5–10 warm up, 30 min heart rate-monitored aerobic, 10–15 min upper- and lowerlimb exercise, 10 min relaxation. Aerobic: 50–60% max HR ≤ 140 bpm	12 weeks	Primary: O2 consumption, Secondary: respiratory exchange ratio, CO2 output, HR, RHR, low birth weight, prematurity, small for gestational age	Not specified	Control: 27.5 ± 2.1, Exercise: 28.0 ± 2.1 (BMI); Baseline weight: control 71.2 ± 7.4, exercise 71.5 ± 7.9 Post-intervention weight: control 77.6 ± 8.3, exercise 77.2 ± 9.1	Exercise group gained approximately 0.5 kg less over 12 weeks, but not statistically significant (p = 0.62). Exercise sessions during pregnancy were not associated with low birth weight 3.363 ± 504 kg (exercise) versus 3.368 ± 518 kg (control), p = 0.97.
Garshasbi et al., 2005^19^	RCT	Midwifesupervised Exercise	3 days/week, 60 min, 5 min slow walking, 5 min extension movements, 10 min general warm-up, 15 min anaerobic, 20 min specific exercise, 5 min return to first position, HR ≤ 140 bpm	12 weeks	Primary: intensity of lowback pain, lordosis, flexibility, maternal weight gain, pregnancy length, neonatal weight	Not specified	Baseline weight: control 55.42 ± 12.90, exercise 67.08 ± 12.8 BMI baseline: control 25.58 ± 5.12, exercise 25.98 ± 4.82 Weight gain during pregnancy: control 13.8 ± 5.2, exercise 14.1 ± 3.8, p = 0.63 Weight of neonate: control 3 500 ± 431 g, exercise 3 426 ± 675 g	No significant difference between two groups according to maternal weight gain and neonatal birth weight. Exercise group gained 0.3 kg more weight
Malpeli et al., 2013^11^	Nonrandomised	Nutritional Intervention	The nutritional intervention consisted of the monthly supply of a basic food basket containing 1 kg fortified wheat flour (30 mg iron, 2 200 μg folic acid, 6.3 mg thiamine, 1.3 mg riboflavin, 13 mg niacin per kg), 2 kg soy-enriched maize flour fortified with micronutrients (1 500 μg RE vitamin A, 8 mg thiamine, 8 mg riboflavin, 100 mg niacin, 1,000 μg folic acid, 40 mg iron, 30 mg zinc per kg), 1 kg sugar, and 1 kg rice. It also contained a nutritional supplement (powder soup, 2 daily servings) equivalent to 250 Kcal daily, 270 μg retinol, 12 μg vitamin D, 20 mg vitamin C, 0.7 mg vitamin B1, 0.7 mg vitamin B2, 0.9 mg vitamin B6, 0.9 μg vitamin B12, 6.8 mg niacin, 200 μg folic acid, 240 mg calcium, 35 mg magnesium, 6 mg iron, 4 mg zinc and 29 mg selenium.	1 year	Weight per trimester, BMI per trimester, low weight, normal weight, overweight, obese, ferritin, iron deficiency (prevalence), folate, prevalence of folate deficiency, zinc, prevalence of zinc deficiency, retinol, prevalence of vitamin A deficiency		At baseline 27.5% were underweight; 25.4% normal weight; 22.4% overweight; 24.7% obese. There was a significant decrease in folate deficiency in the intervention group compared to the control group. The risk of vitamin A deficiency decreased significantly in the intervention group.	No significant differences recorded between intervention and control for anthropometric measurements. Energy and nutrient intake was significantly increased in the intervention group.
Sedaghati et al., 2007^16^	Nonrandomised	Midwife supervised exercise	15 min warm-up and cool-down, 30 min cycling (55–65% MHR), 3 days/week, RPE 12–13,	Not Specified	Intensity of low-back pain, maternal weight gain		Baseline BMI: control 24.30 ± 1.289, exercise 24.10 ± 1.134 Baseline weight: control 61.04 ± 3.681 kg, exercise 60.78 ± 3.577 kg; Weight gain: exercise group 13.55 ± 1.131 kg, control 15.10 ± 2.102 kg, p < 0.0001	Greater increase in weight gain was also seen in the control group
Prevedel et al., 2003^17^	RCT	Aquatic Exercise	Hydrotherapy three times a week. Moderate intensity for 1 hour at a time	Until 36–40 Weeks	Lean body weight (kg), total fat (kg), relative fat (%), VO2 max (ml/kg/min), systolic volume (ml), cardiac output (l/min), full-term/preterm birth, baby’s weight (g)	Not specified	Mean: 58 kg; height: 159–161 cm	No difference in babies’ weight between the two groups (3 175 g control group, 3 110 g intervention group). Significant findings were: the mother’s relative fat percentage increased in the control group but remained the same in the intervention group. Systolic volume and cardiac output increased in the intervention group suggesting better cardiometabolic maternal adaptation.
Cavalcante et al., 2009^18^	RCT	Aquatic Exercise	Water aerobics for 50 min three times a week. Moderate intensity, 24.6 sessions per woman	Until 36 weeks’ gestation	Weight (kg), body fat (%), fat-free mass (%), BMI, % vaginal deliveries, % preterm, neonatal weight	Intervention 63.8 ± 12.7, control 60.8 ± 10.2	Not specified	No significant difference seen between the two groups for any of the outcome measures.
Ghodsi & Asltoghiri, 2012^15^	RCT	Mixed aerobic and flexibility exercise	Mixed exercise regime including stretching and flexibility and aerobic exercises (swimming, cycling, walking) three times a week	20–26th week until delivery	Neonatal weight; 1st and 5th APGAR scale	Not specified	19.8–26 kg/m2 Mean BMI for training group was 23.4 ± 1.9 and 23.3 ± 2.1 for the control Group	No significant difference in neonatal weight between the training and control group (3 204 g vs 3 216 g, respectively). No significant differences in APGAR scale between the two groups. No reporting on maternal weight as an outcome

PA = physical activity; min = minutes; RCT = randomised controlled trial; BMI= body mass index; HT = heart rate; RHR = relative heart rate.

A study by Cavalcante et al.18 also used an intervention of aquatic exercise during pregnancy to determine its effectiveness on maternal outcomes. No differences were noted between control and intervention groups for weight gain during pregnancy, body fat percentage, fat-free mass or body mass index (BMI).

The effects of supervised aerobic exercise on the maternal outcomes of overweight pregnant women were evaluated by Santos et al.^14^ Although oxygen consumption of the exercise group at anaerobic threshold was higher post intervention, neither groups showed any differences in weight change after the intervention.

Two Iranian interventions^16^,^19^ evaluated the effect of landbased exercise on low-back pain during pregnancy. The typical exercise programme for these studies included a combination of midwife-supervised anaerobic and aerobic exercise performed three days per week at a moderate intensity. In the study by Garshabi et al.,^19^ lordosis was reduced in the exercise group after the intervention, but weight gain was similar between the study groups. In addition, spinal flexibility was significantly lower in the exercise group post intervention, and this was correlated with BMI. Weight gain was lower in the control group, and body weight of the neonate was higher than in the exercise group. Although Sedaghati et al.^16^ showed intensity of low-back pain was higher in the control group, weight gain during pregnancy was higher in the exercise group.

The intervention that aimed to determine the possibility of improving maternal outcomes using fortified foods11 found that the prevalence of folic acid and serum retinol deficiency decreased, while vitamin A deficiency remained the same post intervention. No differences were noted for body composition, and the proportions of overweight and obesity in the groups were at a moderate level of 20 and 26.3%, respectively, post intervention.

## Discussion

Pregnancy appears to be a pivotal time for both maternal and foetal health. Emerging research has highlighted the profound effects of the in utero environment on the lifelong health of the baby. More specifically, both underweight and overweight babies are at risk of obesity later on in life.^20^ The perinatal period has been cited by Lawlor and Chaturvedi^21^ as one of the three critical periods in life for the prevention of obesity.

Maternal obesity is perhaps one of the major causes of intrauterine over-nutrition during pregnancy, and can lead to largefor- gestational-age deliveries. In addition, excessive gestational weight gain in both overweight and normal-weight women has been shown to increase obesity in the offspring in both childhood^22^ and adolescence.^23^ For the mother, obesity-related complications and gestational diabetes mellitus may predispose her to the risk of metabolic and vascular diseases later on in life.^24^ Therefore, with the current epidemic of obesity, maternal obesity has serious implications on the health of both current and future generations.

Due to the potential health consequences of maternal obesity, pregnancy is a pivotal period to implement health interventions,^25^ however, little research exists on health promotion during this period.^24^ A previous systematic review by Thangaratinam et al.12 found 44 randomised, controlled trials, conducted in developed countries, which implemented dietary or physical activity interventions to influence maternal weight during pregnancy. In their review, 14 studies implemented physical activity interventions, while 10 looked at dietary interventions and 10 addressed a mixed approach. Similarly, in our study, the majority of interventions focused on physical activity over dietary programmes. It is interesting to note that Thangaratinam et al.^12^ found that diet was more cost effective than physical activity in the management of weight in this population.

The type of interventions used to limit weight gain during pregnancy appears to vary widely between studies. In their systematic review of lifestyle interventions in pregnancy, Oteng-Ntim et al.^26^ found a variety of individual, group and seminar interventions, while in the review by Thangaratinam et al.,^12^ interventions varied from a balanced diet and exercise prescription to counselling and educational sessions. In our study, six out of the seven interventions focused on physical activity, while only one used a nutritional intervention.

Although previous systematic reviews have analysed the literature dealing with interventions during pregnancy for limiting excessive weight gain,^12^,^26^,^27^ ours is the first review of such studies performed solely in developing countries. Changes in diet and activity levels resulting from globalisation and movement of populations from rural to urban environments have led to a rapid rise in the prevalence of obesity in developing countries,^28^ and have caused this disease to move to the top of the public health agenda in many of these countries.^29^

Although there have been calls to focus interventions on maternal nutrition in order to reduce the risk of obesity later on in life,^30^ our review found only seven articles covering maternal obesity interventional studies, with only one specifically addressing nutrition. In addition, although the rate of obesity is high and affects many developing countries, our study showed that only three countries (Brazil, Iran and Argentina) have reported on interventions to curb obesity during pregnancy.

Despite the growing prevalence of obesity in developing countries and the well-recognised detrimental effects of maternal obesity on both maternal and foetal outcomes, this review demonstrates the lack of pertinent research in this area within developing countries. Two of the studies in our review found an increase in weight^16^ and fat percentage^17^ in their control groups, and weight or weight gain was often a secondary outcome measure within the studies reviewed, the majority of which (six of seven studies) were not targeted to overweight women. Other reviews have demonstrated the effectiveness and safety of lifestyle interventions for reducing gestational weight gain,^12^ but this was not strongly demonstrated in the current study.

Key messages:

Lifestyle interventions may be a cost-effective and useful way to manage maternal overweight and obesity as well as gestational weight gain
Few good-quality studies assessing the efficacy of lifestyle interventions on maternal body composition have been conducted in developing countries
In this systematic review of seven studies, two suggested that a physical activity intervention during pregnancy may significantly reduce maternal weight gain; and five were scored as being of poor quality
Future, well-designed lifestyle-intervention studies aimed at managing maternal body composition are much needed in developing countries

Very few studies exist to address the issues of intervention for obesity and weight gain during pregnancy in developing countries. This review summarises the existing literature, of which 71% were of poor quality. Although our review focused on lifestyle interventions for overweight and obesity during pregnancy, the search yielded only one study that aimed the intervention at overweight women, and 50% of the studies were not primarily measuring weight gain as an outcome. In addition, the studies varied significantly from type of intervention to outcome measure, and additionally, the methodology was often poorly described, making comparative and accumulative analysis difficult.

## Conclusion

Dietary and lifestyle interventions during pregnancy may well be the key to addressing the prevention of obesity in future generations.^28^ Physical activity^29^ and dietary^12^ interventions have been shown to play an effective role in maternal weight management in the developed world. To our knowledge, this review is the first to address interventions for weight gain and obesity in developing countries, and few articles appear to have addressed this important issue. Lifestyle interventions may be a cost-effective and useful way to curb the growing epidemic of nutrition-related non-communicable diseases. Despite maternal health and obesity being a public health priority, few robust studies have addressed this critical area.

This review has highlighted the need for further research, and in particular, carefully designed randomised, controlled trials, addressing primarily the issues of weight gain and obesity in pregnancy. Such studies are essential to determine the effectiveness and safety of appropriate lifestyle interventions during pregnancy in resource-limited settings.
